# Supplementary material for: PERADIGM: Phenotype embedding similarity-based rare disease gene mapping
Source: PLoS Genet. 2025 Dec 18;21(12):e1011976. doi: 10.1371/journal.pgen.1011976 (PMC12714201; doi:10.1371/journal.pgen.1011976)
Supplement: S1 Text — Additional methodological details and extended statistical analyses. (PDF) [file pgen.1011976.s001.pdf]

1 Type I Error Rate Control Simulation

To assess the calibration of PERADIGM, we evaluated its type I error control using four complementary analyses based on simulated null p-values. The results are summarized in S 1 Fig and S 2 Table. First, the QQ plots for ADPKD, Q850, and Q874 demonstrate that the observed p-values closely follow the expected null distribution, with no noticeable inflation or deflation across either the bulk or the tail of the distribution. Second, the empirical type I error rates at $\alpha = 0.05$ and $\alpha = 0.01$ (approximately 0.05 and 0.01, respectively) fall within the binomial confidence intervals of the nominal levels, confirming accurate calibration. Third, the Kolmogorov–Smirnov (KS) test p-values (> 0.1) indicate that the overall p-value distributions do not significantly deviate from uniformity. Finally, the genomic inflation factors ($\lambda \approx 1$) further verify that the test statistics are well-calibrated without systematic bias. Together, these results demonstrate that PERADIGM effectively controls the type I error rate across all three evaluated diseases. Although the framework identifies statistically associated genes rather than experimentally validated causal genes, these findings confirm that PERADIGM is statistically robust and that the additional reported genes should be viewed as biologically plausible candidates for future functional investigation.

2 Alternative weighting schemes analysis

As shown in S 2 and S 3 Figs, across the five evaluated weighting schemes, all methods except the IC-only weighting exhibited high pairwise correlations, indicating that they capture largely consistent information patterns among ICD-10 codes. The IC-only scheme differed from the others because it reflects frequency information without incorporating disease-specific association signals. Despite these differences, the overall results from PERADIGM remained highly consistent across weighting strategies. Except for the IC-only scheme, all approaches identified the same set of significant ADPKD-related phenotype–associated genes, demonstrating strong robustness to the choice of weighting metric. Even under the IC-only weighting, causal genes such as *PKD1* and *PKD2* were consistently detected, underscoring the biological stability of the results. Collectively, these findings show that PERADIGM is not sensitive to the specific weighting definition and achieves optimal performance when both phenotype significance and information content are integrated, as each contributes complementary information to the model.

3 Comparison of disease and non-disease group risk score distribution

Our analysis compared the risk scores between disease and non-disease groups for three rare disorders: ADPKD, Marfan syndrome, and NF1 disease. For all three conditions, the disease groups exhibited significantly higher risk scores compared to their respective non-disease counterparts (random sample with equal size as the disease group). This marked difference in risk scores demonstrates the method's robust ability to differentiate between affected and unaffected individuals. These findings underscore the potential of our risk score approach as a valuable tool for distinguishing disease status in rare Mendelian disorders, even within large-scale genomic datasets where such conditions are infrequently represented.
