# Supplementary material for: PERADIGM: Phenotype embedding similarity-based rare disease gene mapping
Source: PLoS Genet. 2025 Dec 18;21(12):e1011976. doi: 10.1371/journal.pgen.1011976 (PMC12714201; doi:10.1371/journal.pgen.1011976)
Supplement: S1 Table — Each row corresponds to a chromosome and reports the number of raw genotype variants, the number of variants retained after quality control, and the number of annotated loss-of-function (LoF) variants. Totals across chromosomes are shown in the final row. (PDF) [file pgen.1011976.s002.pdf]

Table 1. Summary of variants before and after quality control and LoF annotation. Each row corresponds to a chromosome and reports the number of raw genotype variants, the number of variants retained after quality control, and the number of annotated loss-of-function (LoF) variants. Totals across chromosomes are shown in the final row.

Chromosome	Raw Variants	After QC	LoF Variants
Chr1	1,783,906	1,712,567	45,456
Chr2	1,310,712	1,257,244	31,833
Chr3	1,039,941	998,606	26,444
Chr4	717,783	685,611	18,004
Chr5	790,760	757,766	19,752
Chr6	887,003	847,539	23,231
Chr7	858,981	821,664	21,040
Chr8	654,090	627,300	15,079
Chr9	774,556	742,399	17,595
Chr10	733,427	703,066	17,526
Chr11	1,059,628	1,019,090	26,958
Chr12	950,532	910,028	22,992
Chr13	320,631	306,289	8,361
Chr14	557,237	533,269	13,266
Chr15	620,095	594,231	15,532
Chr16	875,192	841,107	19,373
Chr17	1,047,224	1,006,046	25,414
Chr18	286,694	274,147	6,798
Chr19	1,210,652	1,156,885	32,429
Chr20	459,900	441,748	10,394
Chr21	195,726	186,232	4,520
Chr22	414,980	397,963	9,207
**Total**	**17,549,650**	**16,820,797**	**431,204**
