# Supplementary material for: PERADIGM: Phenotype embedding similarity-based rare disease gene mapping
Source: PLoS Genet. 2025 Dec 18;21(12):e1011976. doi: 10.1371/journal.pgen.1011976 (PMC12714201; doi:10.1371/journal.pgen.1011976)
Supplement: S2 Table — Each row reports the genomic inflation factors (λ and λq90), Kolmogorov–Smirnov (KS) test p-values, and empirical type I error rates at α=0.05 and α=0.01, with 95% binomial confidence intervals in parentheses. (PDF) [file pgen.1011976.s003.pdf]

Table 2. Summary of Type I error rate control simulation results. Each row reports the genomic inflation factors (λ and λ_{q90}), Kolmogorov–Smirnov (KS) test p-values, and empirical type I error rates at $\alpha = 0.05$ and $\alpha = 0.01$, with 95% binomial confidence intervals in parentheses. All results demonstrate well-calibrated type I error control across diseases.

**Disease**	λ	λ_{q90}	**KS test p-value**	$\alpha = 0.05$	$\alpha = 0.01$
ADPKD	0.975	1.01	0.426	0.0505 (0.04654–0.0535)	0.0113 (0.00842–0.0116)
Q850	0.989	1.00	0.556	0.0510 (0.04654–0.0535)	0.0111 (0.00842–0.0116)
Q874	0.962	1.00	0.197	0.0513 (0.04654–0.0535)	0.0110 (0.00842–0.0116)
